# Efficacy of Interferential Current Therapy in Patients Diagnosed with Subacromial Impingement Syndrome

**DOI:** 10.5152/eurasianjmed.2023.22282

**Published:** 2023-10-01

**Authors:** Muhammet Tugay, Ayhan Kul

**Affiliations:** Deparment of Physical Medicine and Rehabilitation, Atatürk University Faculty of Medicine, Erzurum, Turkey

**Keywords:** Shoulder impingement syndrome, interferential current, transcutaneous electric nerve stimulation

## Abstract

**Objective:**

The objective of the study was to evaluate the effectiveness of interferential current treatment on a range of motion of joint and shoulder pain, functional status, and quality of life in patients with subacromial impingement syndrome and to compare interferential current with transcutaneous electrical nerve stimulation and sham interferential current.

**Materials and Methods:**

Patients complaining of shoulder discomfort participated in the present study. Diagnosis of subacromial impingement syndrome is based on anamnesis, clinical examinations, and shoulder magnetic resonance imaging. A total of 52 patients divided into 3 groups: Group 1 (17 patients, mean age 51.8 years) received interferential current, group 2 (18 patients, mean age 51.8 years) received transcutaneous electrical nerve stimulation, and group 3 (17 patients, mean age 49.1 years) received sham interferential current. Hot pack and exercise treatments were added to all groups. All groups were treated for 3 weeks, 5 times a week, for 15 sessions and 20 minutes for each session. Evaluations were made before treatment (T0), in the middle of treatment (T1; end of 8th session), and at the end of treatment (T2; end of 15th session) using active range of motion and visual analog scale for pain, the Arm, Shoulder, and Hand Problems Questionnaire for functional status, and Short Form-36 for quality of life.

**Results:**

There were significant improvement effects on all of the range of motion, visual analog scale, and the Arm, Shoulder, and Hand Problems Questionnaire scores at T2 and on the scores in some subparameters of Short Form-36 in all groups (*P* < .05). However, there was no statistically significant difference at T2 between the groups (*P* > .05).

**Conclusion:**

Interferential current and transcutaneous electrical nerve stimulation exhibited equivalent results regarding range of motion, pain, function, and quality of life of patients with subacromial impingement syndrome, with no significant difference between interferential current and transcutaneous electrical nerve stimulation. Adding interferential current or transcutaneous electrical nerve stimulation treatments to hot pack +exercise therapy did not result in any extra benefits to the patients.

**Main Points**

- Only 1 study investigated the therapeutic efficacy of IFA treatment on subacromial impingement syndrome (SIS).
- IFA, transcutaneous electrical nerve stimulation, and S-İFA treatment protocols were effective in patients with SIS. However, we found no superiority of 1 protocol compared to other(s).
- Only hot pack and exercise therapy would be sufficient.

## Introduction

The term “subacromial impingement syndrome” (SIS) refers to a collection of shoulder symptoms and physical and radiographic results that may be attributed to the compression of tissues surrounding the glenohumeral joint. This might be done by raising the afflicted shoulder. This disorder, which includes subacromial space pathologies such as rotator cuff partial tears, rotator cuff tendinitis, calcific tendinitis, and subacromial bursitis, is the most prevalent cause of shoulder discomfort and dysfunction in everyday and occupational life.^1,2^ To stop the inflammatory process, alleviate pain, preserve joint mobility, and prevent progressive pathological changes, SIS uses a variety of physical therapy applications, including prevention, rest, medical treatment, exercises, and electrotherapy modalities.^2^

Electrotherapy methods are essential in treating shoulder pain pathologies due to their analgesic, anti-inflammatory, and local vasodilator effects. Transcutaneous electrical nerve stimulation (TENS), a low-frequency electrical current, is widely used in SIS and other musculoskeletal system (MSS) pathologies due to its ease of use and portability.^3^ Transcutaneous electrical nerve stimulation is classified as an electro-analgesic resource and is based on the application of alternating medium-frequency current (4000 Hz) with amplitude modulation at low frequency (0-250 Hz) (0-250 Hz).^4^ Interferential current (IFC) therapy provides more effective analgesia for the patient in the targeted tissue by penetrating more effectively and more deeply in the targeted treatment area due to its ability to reduce skin impedance.^5^ In addition, another advantage over TENS is its ability to provide lower neural adaptation. For these reasons, it is used in the treatment of SIS and other MSS pathologies.^3,4^

A limited number of studies evaluated the effectiveness of IFC treatment in patients with SIS and compared it with other physical therapy treatment methods.^3,4,6,7^ A few studies have compared the effectiveness of IFC and TENS treatments. Ucurum et al^3^ evaluated IFC and TENS with no sham-treated control group and found no statistically significant difference in the 2 therapies’ effectiveness.

We aimed to evaluate the effectiveness of IFC treatment on a range of motion (ROM) of joint and shoulder pain, functional status, and quality of life in patients with SIS and to compare IFC with TENS and sham IFC (S-IFC).

## Materials and Methods

This prospective, randomized, controlled clinical study was conducted between July 2020 and July 202 in the AtatürkUniversity, Faculty of Medicine, Department of Physical Medicine and Rehabilitation.

Patients who participated in the study were informed of its objective, duration, method of administration, adverse effects, and potential complications. In addition, all patients signed a document requesting their informed consent. This research was approved by the Ethics Committee of Atatürk University, Faculty of Medicine (June 24, 2020/7; 61). The study was conducted in conformity with the Helsinki Declaration’s standards.

Patients who applied to the Department’s polyclinic with the complaint of shoulder discomfort were considered for participation in the study. After examination by anamnesis, clinical examination, and shoulder magnetic resonance imaging, the patients have been diagnosed with SIS (MRI). The participation of 52 patients in the trial was entirely voluntary.

Neer, Hawkins, painful arc, drop arm, Yergason, supraspinatus, and active ROM tests were performed for clinical diagnosis. Patients were enrolled in the study if they met the following criteria: (1) they were diagnosed with subacute or chronic SIS clinically and radiologically (diagnosed by a radiologist on MRI); (2) they were aged between 18 and 65 years; and (3) they had tendinitis or a partial rupture in the supraspinatus.

Patients who had adhesive capsulitis or bicipital tendinitis, total rupture of the rotator cuff or supraspinatus muscle, undergone shoulder joint operation, inflammatory disease in the shoulder region, cervical radiculopathy, metabolic bone diseases, and diabetes mellitus were excluded from the study. In addition, patients who had received conservative physical therapy to the shoulder within the past 6 months and a local steroid injection treatment within the past 3 months were also excluded.

Patients eligible for the study were given a number corresponding to the sequence in which they were admitted. Then the patients were randomly assigned to 1 of 3 groups using an online randomization program (randomizer.org). Hot pack + transcutaneous electrical nerve stimulation was administered to the first group, HP + IFC to the second group, and HP + S-IFC to the third group (Intelect Advanced, 2762CC model, Chattanooga Group, Hixson, Tennessee, United States). Throughout the therapy period, therapeutic shoulder exercises were administered to all groups. Before the treatment (T0; on day 0), in the middle of the treatment (T1; at the conclusion of the 8th session), and after the treatment were utilized to collect data for the assessment of the patients (T2; at the end of the 15th session).

Evaluations were made before the treatment (T0, day 0), in the middle of the treatment (T1; end of 8th session), and at the end of the treatment (T2; end of 15th session) using active ROM and visual analog scale (VAS) for pain, the Arm, Shoulder, and Hand Problems Questionnaire (DASH) for functional status, and Short Form-36 (SF-36) for quality of life.

Using a conventional goniometer, the active shoulder ROM was measured. Active shoulder ROM measures are restricted in SIS patients, particularly during abduction and internal rotation. Since shoulder motions exacerbate compression and discomfort, patients avoid arm movement in these directions.^8^ Therefore, we examined ROM values during the active flexion, abduction, and internal rotation.

The scale is called VAS, and it has a 10 cm character. Patients were asked to identify the average level of pain they had experienced during the previous week, with “0” indicating no pain, “5” denoting mild discomfort, and “10” denoting the most severe pain. The VAS values for rest (VAS-R), activity (VAS-A), and night were determined (VAS-N).^9^

The DASH questionnaire has been approved for use in certain arm disorders. The patients responded to all inquiries using the proper 5-point Likert scale (1: no difficulty, 2: mild difficulty, 3: moderate difficulty, 4: extreme difficulty, 5: inability to do it at all). Scores range from 0 to 100 (0: no disability, 100: maximum disability).^10^

The SF-36 is a short-form, multipurpose health questionnaire with just 36 items. It provides a profile with a score on 8 scales and brief, concise measurements of physical and mental health. Physical function (10 items), physical role (4 items), body pain (2 items), general health (6 items), vitality (4 items), social function (2 items), emotional role (3 items), and mental health make up the SF-36’s 8 subscales and 36 questions (5 items). Short Form-36 ratings vary from 0 to 100, with higher scores indicating more excellent health. The scale is examined in light of the previous 4 weeks.^11^

To minimize muscular spasms, all patients had 15 sessions of 20-minute H-P 5 times per week for 15 weeks. After this warm-up and relaxation period, electrotherapy was administered to the patients. In group 1, 4 electrodes were inserted across the glenohumeral joint with the glenohumeral joint in the center. Then, a 20-minute IFC therapy was administered, producing an amplitude of 80-150 Hz. In group 2, the painful shoulder region was treated with TENS using an active electrode with a pulse length of 20-60 μs and a stimulation frequency of 95 Hz.

There were fifteen 30-minute sessions each day, 5 days per week. In group 3, the electrodes and patient location were modified according to the IFC technique. The S-IFC therapy was administered by turning on the device’s lights in the same manner, shutting off the device without supplying electricity, and emitting the end signal sound. The same physiotherapist did active–passive ROM, stretching (posterior capsule), Codman, wheel, finger chart, and isometric strengthening exercises following each session. In addition, patients were given an illustration of the exercises for use at home. Each exercise had to be performed 3 times each day, at least 10 times. The identical workout program was administered to all 3 groups. In addition, the patients were given instructions, such as avoiding overhead activities, for the relative rest of the affected shoulder. The patients’ adherence to exercise was checked daily by telephone before and after therapy. There were no complications identified among the patients. During the study, no analgesics were authorized (except paracetamol, as required). Paracetamol intake was also prohibited before measurements. Diagnosis and evaluations of the patients were made by 2 authors of the study (M.T and A.K.).

### Statistical Analysis

The Statistical Package for Social Sciences v.22 for Windows (IBM SPSS Corp.; Armonk, NY, USA) was used to analyze the study’s collected data. The sample size estimation is based on the findings of a previous study^3^: the pretreatment VAS-R (mean SD: 3.05) and the posttreatment VAS-R third months (mean SD: 1,58) values (effect size = 0.793) were calculated with the G power program, and it was determined that there needed to be at least 19 patients included in the study to achieve 95% CI and 93% power level in the groups. We collected various general descriptive statistics, including the mean, median, and SD values of continuous variables. The Shapiro–Wilk and Kolmogorov–Smirnov tests were used to evaluate the normalization of the numerical data’s distribution. The chi-square or Fisher analysis was used to conduct the discrete distribution study between the groups. “*t*-test between the 2 groups” was the statistical analysis performed on data exhibiting normal distribution. Analyzing the differences between the 2 independent groups of continuous variables required the application of the Mann–Whitney *U*-test. This test was applied to the data with a non-normal distribution. The “paired *t*-test” was used to analyze data that exhibited normal distribution. The Wilcoxon test was used for the data showing a non-normal distribution to analyze the disparities between 2 dependent groups of continuous variables. A comparison was made between the Kruskal–Wallis test, used for non-normally distributed data, and the analysis of variance, used for regularly distributed data in more than 2 independent groups. The repeated measures analysis of variance for normally distributed data was contrasted with the Friedman test, which was used for non-normally distributed data, in the context of the group comparison of repeated measurements. It was established by applying the post hoc tests as well as the Wilcoxon tests on the various groups. The significance level of the findings was determined to be *P* = .05, and the CI was calculated to be 95%.

## Results

**Figure 1.**
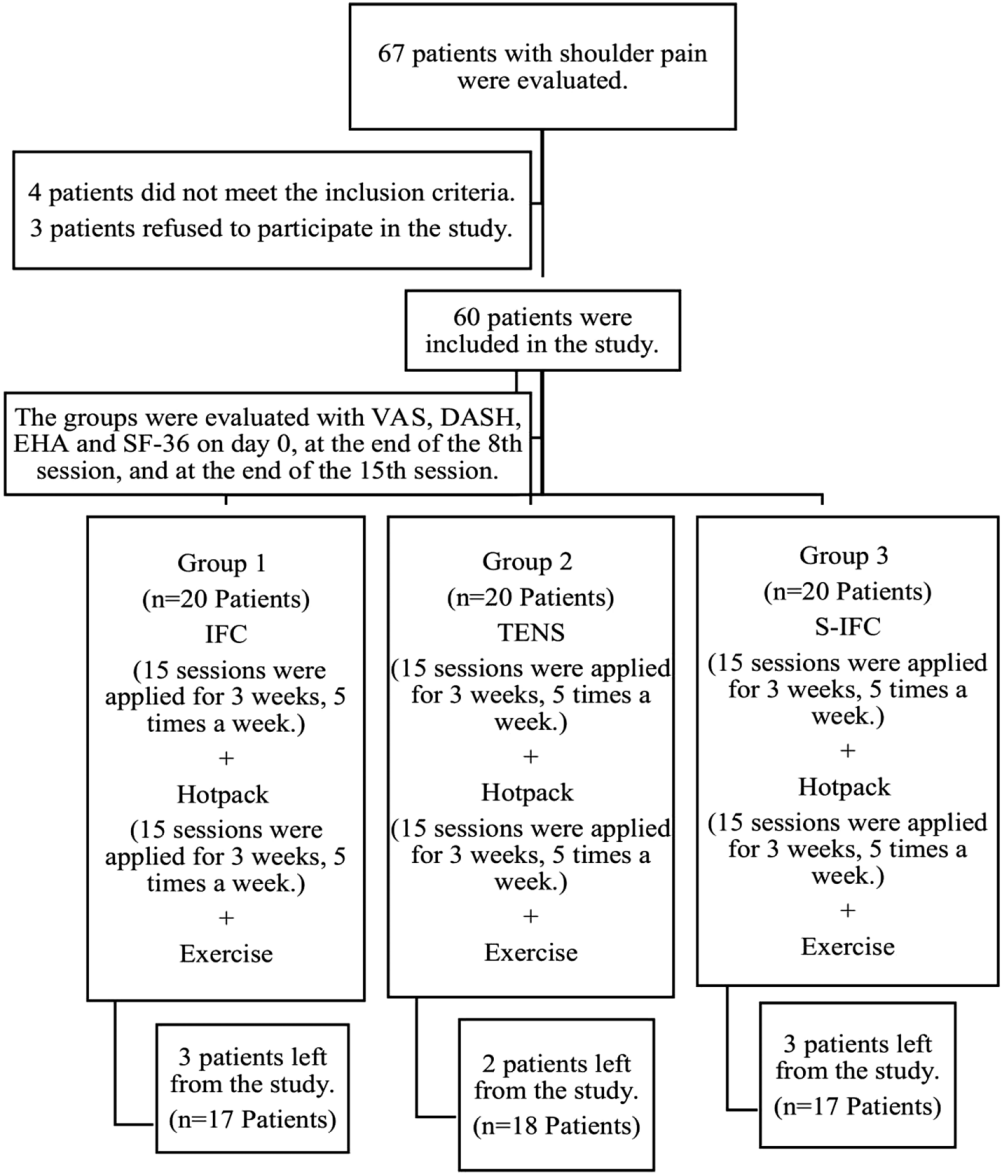
Working flowchart.

At the beginning of the research, there were 67 patients in the sample group. Because they did not meet the diagnostic requirements, 4 patients were disqualified from participation in the study. Later on, 3 patients from group 1, 2 from group 2, and 3 from group 3 dropped out of the study; hence, these patients were not included in the sample for the research. The whole sample consisted of 52 patients (Figure 1). Table 1 presents the demographic and clinical information of the groups for your perusal. The demographic and clinical data of the groups did not significantly vary from one another (*P *> .05) (Table 1).

Table 2 presents the findings from investigating the various parameters used to assess the treatment’s effectiveness in the 3 groups.

Shoulder abduction, VAS-N, DASH, and SF-36 (vitality) subparameter scores showed significant improvements in the T0-T1 period in the IFC group. This improvement was also obtained at flexion, VAS-A, VAS-R, and DASH scores in the T1-T2 period, and at flexion, abduction, internal rotation, and VAS-A, VAS-N, VAS-R, and DASH scores in the T0-T2 period in this group (Table 2).

Abduction, VAS-A, VAS-R, VAS-N, and DASH scores showed significant improvements at T0-T1 period in the TENS group. The VAS-N scores in the T1-T2 period showed significant improvement. Flexion, abduction, internal rotation, VAS-A, VAS-N, VAS-R, and DASH scores in the T0-T2 period, as well as from body pain score, which is the SF-36 (Table 2) showed significant improvements.

In the S-IFC group, significant improvement was obtained from scores for flexion, abduction, VAS-N, and DASH in the T0-T1 period; from VAS-A in the T1-T2 period; and from scores for flexion, abduction, internal rotation, VAS-A, VAS-N, VAS-R, and DASH in the T0-T2 period; as well as from the body pain score, which is the SF-36 subparameter [*P* (Table 2)].

Table 3 compares the ROM, VAS, and DASH scores across the groups based on the T0, T1, and T2 assessment outcomes. The scores on the ROM, VAS, and DASH questionnaires did not vary significantly across the groups (*P* > .05) (Table 3). As a result, there was a statistically significant change in the overall health perception score (which is one of the SF-36 subparameters) in the T2 period only in the TENS group when compared to the scores of the other groups (*P* < .05).

## Discussion

According to the findings of our research, all of the IFC, TENS, and S-IFC treatments, in addition to the HP+ exercise therapy for SIS, had significant curative effects (T1) by demonstrating their effectiveness on a ROM, pain, function, and quality of life in a relatively short amount of time. In addition, there were statistically significant improvements in all of the ROM, pain, and function values that were examined at the conclusion of the therapy (T2) in all of the groups. Furthermore, in terms of quality of life, some substantial improvements were noted in the scores of many other quality-of-life subparameters. On the other hand, there was not a discernible difference between the groups after therapy had been completed (T2).

Subacromial impingement syndrome is the most prevalent cause of shoulder discomfort. The goal of conservative therapy for SIS is to enhance function and quality of life while reducing pain, discomfort, and inflammation in the subacromial space. Anti-inflammatory medicines, superficial thermal treatments (cold pack and HP), exercise therapy, and electrotherapy modalities are only some of the many therapeutic possibilities available. However, the most effective therapy for SIS is still a contentious topic of debate.^12^ Nowadays, therapeutic exercises are accepted as an effective treatment method in terms of pain, function, and quality of life.^13-15^ In addition, Granviken et al^16^ determined that both indoor and supervised exercises showed similarly effective improvement in pain and function in the treatment of SIS. During our study, HP and an identical exercise treatment were administered to all groups using various electrotherapy techniques. The applied exercise program consisted of activities that were carried out both indoors and while being supervised. As a result, the groups considerably reduced their pain and dysfunction levels. However, there was no discernible change in ROM, pain levels, functional ability, or quality of life between the S-IFC group, which served as a control, and the groups treated with IFC and TENS. In this light, it was concluded that the exercise therapy we employed in the treatment of SIS was successful, and the findings of our study were consistent with the findings of the other research. In addition, it has been shown in the study that prolonged physical activity benefits the overall quality of life following therapy.^17^ Exercise therapy was the only treatment administered in this research project’s group (HP + exercise + S-IFC), which showed a substantial improvement in the bodily pain subcomponent of quality of life compared to the previous condition state. The lack of progress in other aspects may be attributable to the fact that our study was just 15 sessions long, and there needed to be a long-term follow-up.

It is still unclear how many electrotherapeutic compounds alleviate pain, boost mobility, and prepare tissues for activity work.^18-21^ In our study, several electrotherapeutic agents, such as IFC, TENS, and S-IFC, were applied to the affected areas of the participants in conjunction with high-pressure and exercise treatment. Interferential current treatment is a kind of electrical stimulation based on 2 medium-frequency alternating currents of different frequencies. This impacts deep tissues like a low-frequency current, a method often used in electrotherapy. On the other hand, more data about its efficiency need to be collected.^4^ A systematic review and meta-analysis of Fuentes et al^4^ studied the efficacy of IFC treatment in musculoskeletal pain. The authors reported that the IFC therapy, a multimodal treatment, may be successful in terms of analgesics in acute or chronic painful situations in various musculoskeletal disorders. This is compared to individuals who do not get any treatment or receive a placebo. However, these authors also found that IFC therapy was not substantially superior to a placebo or other therapies at the study’s discharge or the follow-up stages. In addition, Van der Heijden et al^22^ compared exercise therapy alone (active and passive ROM of the shoulder) to IFC and placebo IFC in addition to exercise therapy in the treatment of soft tissue disorders in the shoulder; as a result of this, they reported that there was no difference between the groups in terms of pain and functional capacity in short- and long-term follow-ups; therefore, there was no benefit of adding IFC therapy as an adjuvant to exercise therapy. Our study found no significant difference between the 2 groups; however, the groups who got exercise therapy in addition to IFC or S-IFC treatments exhibited substantial improvement in ROM, discomfort, function, and overall quality of life. As a result, the IFC treatment combined with HP and exercise therapy did not result in any further benefits for a ROM), discomfort, function, or quality of life. On the other hand, our investigation did not include a separate IFC group, which may be a limitation of our study. This may be why we could only get limited information regarding the efficiency of the IFC therapy.

The TENS treatment is one of the most common applications utilized in shoulder tendon diseases because it improves blood circulation and prepares the patient for activity by lowering discomfort.^3^ One of the primary hypotheses for the mechanism of action of analgesics is based on the gate-control theory. According to this theory, peripheral inhibition of pain can be obtained as a result of stimulation of large non-nociceptive afferent fibers. This hypothesis is one of the primary hypotheses for the mechanism of action of analgesics. Transcutaneous electrical nerve stimulation offers patients several benefits, the most notable of which are that it is inexpensive, secure since it has few adverse effects, and easy for patients to use on their own.^23,24^ In a recent randomized controlled study that examined the efficacy of TENS therapy on pain and function in rotator cuff tendinopathy, it was shown that TENS application is an effective modality. The study was carried out to investigate the efficiency of TENS treatment.^25^ In this context, many studies studied the effects of TENS treatment, and they found it an effective treatment method for pain and function in many musculoskeletal pathologies (lateral epicondylitis, neck pain, etc.).^26,27^ However, as a result of the review studies of Desmeules et al^19^ in which they examined the effectiveness of TENS in rotator cuff tendinopathies, they reported that due to the high bias in the studies included in the review, a conclusion could not be reached about the effectiveness of TENS, that better quality studies are needed methodologically, and that until then, clinicians should prefer other evidence-based rehabilitation practices that have proven to be effective in treating patients with rotator cuff tendinopathy. In addition, similar findings regarding the efficacy of TENS therapy for shoulder pain were validated owing to the methodological constraints of current studies as a result of a Cochrane review that evaluated TENS treatment for shoulder pain.^28^ In our study, it was discovered that the other groups did not vary. However, the group that had TENS treatment in addition to exercise therapy showed considerable improvement in terms of ROM, discomfort, function, and quality of life. Therefore, it was determined that adding TENS treatment to HP + exercise therapy did not provide any further benefits for ROM, pain, function, or quality of life. This result was consistent with the findings of previous large-scale Cochrane studies. However, the lack of the S-TENS group in our research may be regarded as a limitation.

We compared the efficacy of individual IFC and TENS treatments for SIS. In the study by Ucurum et al,^3^ it was determined that there was a significant improvement in the physical and mental components of pain, function, and quality of life 4 weeks after IFC treatment and a similar improvement in the physical component of pain, function, and quality of life after TENS treatment. When the treatments were compared, it was found that there was no difference between them in terms of pain, function, and the physical component of quality of life and that they showed a similar improvement. In addition, it was determined that there was no difference between them regarding the physical component of quality of life. However, the authors pointed out that this study was flawed because a sham group was included. Regarding our research, having an additional S-IFC group in addition to the groups that were treated with IFC and TENS in a manner comparable to the study that came before ours may be a potential benefit. It was shown that the effects of each group on a ROM, pain, and function were comparable to the research presented before. While an improvement was seen in scores for physical function, vitality, body pain, and general health perception, all of which are subcomponents of quality of life, especially after IFC treatment, a significant improvement was seen only in the subcomponent of body pain in the TENS and S-IFC groups. This was the only subcomponent of quality of life that significantly improved. On the other hand, when the quality of life of each group was compared to one another, it was found that the only subcomponent of quality of life that differed significantly in favor of the TENS group was the overall health perception subcomponent. When the findings of our research are analyzed, it is found that the combination of HP and exercise therapy can be effective on a ROM, pain, function, and quality of life; however, the addition of IFC or TENS therapy in addition to exercise therapy does not provide a significant advantage. Therefore, it demonstrates that more placebo-controlled, randomized, controlled studies involving IFC and TENS therapy are still required. These studies should also have a longer follow-up period, a greater number of patients, and improved methodology and should be conducted independently of HP and exercise therapy. Consequently, clearer views can be put forth in the light of investigations concerning the effects of IFC and TENS therapies, how much and how their effects are, and how their effects are.

Due to the short follow-up period, the long-term efficacy of the treatments could not be evaluated. Additionally, the results could not be examined in a larger population due to the low patient participation in our study during the coronavirus disease-2019 pandemic. Other possible limitations of our study include the inability to compare the efficacy of electrotherapy modalities alone with the sham groups and the inability to form a group that was not treated due to ethical problems.

## Conclusion

Interferential current and TENS exhibited equivalent results regarding the ROM, pain, function, and quality of life of patients with SIS, with no significant difference between IFC and TENS. Adding IFC or TENS treatment to HP + exercise therapy did not result in any extra benefits to the patient.

## Figures and Tables

**Table 1.** Demographic and Clinical Characteristics of the Groups

Variables	Group 1 IFC (n = 17), Mean ± SD	Group 2 TENS (n = 18), Mean ± SD	Group 3 S-IFC (n = 17), Mean ± SD	*P*
Age (years)	51.8 ± 8.8	51.8 ± 12.1	49.1 ± 12.3	.716
BMI (kg/m^2^)	29.1 ± 3.3	30.9 ± 6.7	28.9 ± 3.7	.908
Gender; n (%)				
Male	4 (23.5%)	3 (16.7%)	2 (11.8%)	.660
Female	13 (76.5%)	15 (83.3%)	15 (88.2%)	
Disease duration (months)	9.9 ± 10.6	9 ± 8.6	5.9 ± 8.2	.170
Dominant side				
Right, n (%)	16 (94.1%)	17 (94.4%)	15 (88.2%)	.639
Left; n (%)	1 (5.9%)	1 (5.6%)	2 (11.8%)	
Sick side				
Left; n (%)	8 (47.1%)	8 (44.4%)	5 (29.4%)	.518
Right; n (%)	9 (52.9%)	10 (55.6%)	12 (70.6%)	

*P* < .05: statistically significant difference between the groups.

BMI, body mass index; IFC, interferential current; S-IFC, sham interferential current; TENS, transcutaneous electrical nerve stimulation.

**Table 2.** Intragroup Comparison of ROM, VAS, and DASH scores in T0, T1, and T2

Variables	Group	T0, Mean ± SD	T1, Mean ± SD	T2, Mean ± SD	*P*
ROM (active)					
Flexion	IFC	131.5 ± 27.2	143.2 ± 25.6	157.9 ± 19.6	<.001^b,c^
Flexion	TENS	128.1 ± 36.9	149.7 ± 30.8 155	160.3 ± 26.6	<.001^b^
Flexion	S-IFC	120.3 ± 23.7	141.5 ± 28.9	150 ± 27.3	<.001^a,b^
Abduction	IFC	121.2 ± 34.6	133.2 ± 28.6	149.1 ± 25.8 155	<.001^a,b^
Abduction	TENS	120.3 ± 31.2	149.2 ± 25.2	163.1 ± 28.3	<.001^a,b^
Abduction	S-IFC	118.8 ± 26	139.1 ± 29.1	146.8 ± 29.6 155	<.001^a,b^
Internal rotation	IFC	80.6 ± 9.5	79.7 ± 12.1	85.6 ± 7.3	.027^b^
Internal rotation	TENS	75.3 ± 13.4	79.7 ± 10.4	84.4 ± 7.3	<.001^b^
Internal rotation	S-IFC	76.8 ± 13.1	83.2 ± 10.3	84.7 ± 9.9	.023^b^
VAS					
VAS-A	IFC	8.3 ± 1.6	7.1 ± 1.5	4.6 ± 2.2	<.001^b,c^
VAS-A	TENS	7.1 ± 1.5	5.9 ± 1.4	4.9 ± 1.4	<.001^a,b^
VAS-A	S-IFC	6.6 ± 2.2	6.2 ± 2.1	5.4 ± 2.1	.002^b,c^
VAS-R	IFC	6.8 ± 1.9	5.7 ± 2.2	3.8 ± 1.9	<.001^b,c^
VAS-R	TENS	5.8 ± 1.5	4.4 ± 1.2	3.6 ± 1.3	<.001^a,b^
VAS-R	S-IFC	5.5 ± 0.8	4.6 ± 1.5	3.5 ± 2	<.001^b^
VAS-N	IFC	7.9 ± 2.3	6.2 ± 2.4	4.4 ± 2.3	<.001^a,b^
VAS-N	TENS	7.2 ± 2	5.7 ± 2.4	4.3 ± 2.1	<.001^a,b,c^
VAS-N	S-IFC	8.4 ± 1.7	6.5 ± 1.9	5.4 ± 2.2	.001^a,b^
DASH					
DASH	IFC	62 ± 15.1	51.8 ± 15.9	41.3 ± 17.1	<.001^a,b,c^
DASH	TENS	59.3 ± 13.5	49.1 ± 14.1	42.9 ± 16.6	<.001^a,b^
DASH	S-IFC	60.6 ± 13.1	52.1 ± 19.4	48.3 ± 17.4	.001^a,b^

^a^Statistical difference between the end of the day 0 and the session 8.

^b^Statistical difference between day 0 and the session 15.

^c^Statistical difference between the 8th session and the 15th session.

*P* < .05: statistically significant difference between the groups.

DASH, The Disabilities of the Arm, Shoulder, and Hand; IFC, interferential current; ROM, range of motion of joint; S-IFC, sham interferential current; T0, before treatment (day 0); T1, in the middle of treatment (end of eighth session); T2, after treatment (end of 15th session); TENS, transcutaneous electrical nerve stimulation; VAS-A, visual analog scale—activity; VAS-R, visual analog scale—rest; VAS-N, visual analog scale—night.

**Table 3.** Intergroup Comparison of ROM, VAS, and DASH scores at T0, T1, and T2

Variables	Group 1 IFC (n = 17), Mean ± SD	Group 2 TENS (n = 18), Mean ± SD	Group 3 S-IFC (n = 17), Mean ± SD	*P*
ROM (Active)				
Flexion: T0	131.5 ± 27.2	128.1 ± 36.9	120.3 ± 23.7	.072
Flexion: T1	143.2 ± 25.6	149.7 ± 30.8	141.5 ± 28.9	.575
Flexion: T2	157.9 ± 19.6	160.3 ± 26.6	150 ± 27.3	.379
Abduction: T0	121.2 ± 34.6	120.3 ± 31.2	118.8 ± 26	.133
Abduction: T1	133.2 ± 28.6	149.2 ± 25.2	139.1 ± 29.1 150	.526
Abduction: T2	149.1 ± 25.8	163.1 ± 28.3	146.8 ± 29.6 155	.085
Internal rotation: T0	80.6 ± 9.5	75.3 ± 13.4	76.8 ± 13.1	.480
Internal rotation: T1	79.7 ± 12.1	79.7 ± 10.4	83.2 ± 10.3	.546
Internal rotation: T2	85.6 ± 7.3	84.4 ± 7.3	84.7 ± 9.9	.783
VAS				
VAS-A: T0	8.3 ± 1.6	7.1 ± 1.5	6.6 ± 2.2	.270
VAS-A: T1	7.1 ± 1.5	5.9 ± 1.4	6.2 ± 2.1	.088
VAS-A: T2	4.6 ± 2.2	4.9 ± 1.4	5.4 ± 2.1	.290
VAS-R: T0	6.8 ± 1.9	5.8 ± 1.5	5.5 ± 0.8	.126
VAS-R: T1	5.7 ± 2.2	4.4 ± 1.2	4.6 ± 1.5	.154
VAS-R: T2	3.8 ± 1.9	3.6 ± 1.3	3.5 ± 2	.775
VAS-N: T0	7.9 ± 2.3	7.2 ± 2	8.4 ± 1.7	.135
VAS-N: T1	6.2 ± 2.4	5.7 ± 2.4	6.5 ± 1.9	.773
VAS-N: T2	4.4 ± 2.3	4.3 ± 2.1	5.4 ± 2.2	.835
DASH				
DASH: T0	62 ± 15.1	59.3 ± 13.5	60.6 ± 13.1	.988
DASH: T1	51.8 ± 15.9	49.1 ± 14.1	52.1 ± 19.4	.343
DASH: T2	41.3 ± 17.1	42.9 ± 16.6	48.3 ± 17.4	.855

*P* < .05: statistically significant difference between the groups.

DASH, The Disabilities of the Arm, Shoulder, and Hand; IFC, interferential current; ROM, range of motion of joint; S-IFC, sham interferential current; T0, before treatment (day 0); T1, in the middle of treatment (end of eighth session); T2, after treatment (end of 15th session); VAS-A, visual analog scale—activity; VAS-R, visual analog scale—rest; VAS-N, visual analog scale—night; TENS, transcutaneous electrical nerve stimulation.

## References

[1] Larsson R Bernhardsson S Nordeman L . Effects of eccentric exercise in patients with subacromial impingement syndrome: a systematic review and meta-analysis. BMC Musculoskelet Disord. 2019;20(1):446. (10.1186/s12891-019-2796-5) PMC6792214 31610787

[2] Badıl Güloğlu S . Comparison of low-level laser treatment and extracorporeal shock wave therapy in subacromial impingement syndrome: a randomized, prospective clinical study. Lasers Med Sci. 2021;36(4):773 781. (10.1007/s10103-020-03093-0) 32638239

[3] Gunay Ucurum S Kaya DO Kayali Y Askin A Tekindal MA . Comparison of different electrotherapy methods and exercise therapy in shoulder impingement syndrome: a prospective randomized controlled trial. Acta Orthop Traumatol Turc. 2018;52(4):249 255. (10.1016/j.aott.2018.03.005) 29703659 PMC6150449

[4] Fuentes JP Armijo Olivo S Magee DJ Gross DP . Effectiveness of interferential current therapy in the management of musculoskeletal pain: a systematic review and meta-analysis. Phys Ther. 2010;90(9):1219 1238. (10.2522/ptj.20090335) 20651012

[5] Gundog M Atamaz F Kanyilmaz S Kirazli Y Celepoglu G . Interferential current therapy in patients with knee osteoarthritis: comparison of the effectiveness of different amplitude-modulated frequencies. Am J Phys Med Rehabil. 2012;91(2):107 113. (10.1097/PHM.0b013e3182328687) 22019968

[6] Nazligul T Akpinar P Aktas I Unlu Ozkan F Cagliyan Hartevioglu H . The effect of interferential current therapy on patients with subacromial impingement syndrome: a randomized, double-blind, sham-controlled study. Eur J Phys Rehabil Med. 2018;54(3):351 357. (10.23736/S1973-9087.17.04743-8) 28895673

[7] Gomes CAFP Dibai-Filho AV Moreira WA Rivas SQ Silva EDS Garrido ACB . Effect of adding interferential current in an exercise and manual therapy program for patients with unilateral shoulder impingement syndrome: a randomized clinical trial. J Manipulative Physiol Ther. 2018;41(3):218 226. (10.1016/j.jmpt.2017.09.009) 29459121

[8] Kul A Ugur M . Comparison of the efficacy of conventional physical therapy modalities and Kinesio taping treatments in shoulder impingement syndrome. Eurasian J Med. 2019;51(2):139 144. (10.5152/eurasianjmed.2018.17421) 31258353 PMC6592440

[9] Wang JC Chang KV Wu WT Han DS Özçakar L . Ultrasound-guided standard vs dual-target subacromial corticosteroid injections for shoulder impingement syndrome: a randomized controlled trial. Arch Phys Med Rehabil. 2019;100(11):2119 2128. (10.1016/j.apmr.2019.04.016) 31150601

[10] Pekgöz F Taşkıran H Kaya Mutlu E Atalay A Çeliker R . Comparison of mobilization with supervised exercise for patients with subacromial impingement syndrome. Turk J Phys Med Rehabil. 2020;66(2):184 192. (10.5606/tftrd.2020.3649) 32760896 PMC7401680

[11] Yılmaz M Eroglu S Dundar U Toktaş H . The effectiveness of high-intensity laser therapy on pain, range of motion, functional capacity, quality of life, and muscle strength in subacromial impingement syndrome: a 3-month follow-up, double-blinded, randomized, placebo-controlled trial. Lasers Med Sci. 2022;37(1):241 250. (10.1007/s10103-020-03224-7) 33400012

[12] Dong W Goost H Lin XB , et al. Treatments for shoulder impingement syndrome: a PRISMA systematic review and network meta-analysis. Med (Baltim). 2015;94(10):e510. (10.1097/MD.0000000000000510) PMC4602475 25761173

[13] Letafatkar A Rabiei P Kazempour S Alaei-Parapari S . Comparing the effects of no intervention with therapeutic exercise, and exercise with additional Kinesio tape in patients with shoulder impingement syndrome. A three-arm randomized controlled trial. Clin Rehabil. 2021;35(4):558 567. (10.1177/0269215520971764) 33155484

[14] Ravichandran H Janakiraman B Gelaw AY Fisseha B Sundaram S Sharma HR . Effect of scapular stabilization exercise program in patients with subacromial impingement syndrome: a systematic review. J Exerc Rehabil. 2020;16(3):216 226. (10.12965/jer.2040256.128) 32724778 PMC7365732

[15] Gutiérrez-Espinoza H Araya-Quintanilla F Cereceda-Muriel C Álvarez-Bueno C Martínez-Vizcaíno V Cavero-Redondo I . Effect of supervised physiotherapy versus home exercise program in patients with subacromial impingement syndrome: A systematic review and meta-analysis. Phys Ther Sport. 2020;41:34 42. (10.1016/j.ptsp.2019.11.003) 31726386

[16] Granviken F Vasseljen O . Home exercises and supervised exercises are similarly effective for people with subacromial impingement: a randomised trial. J Physiother. 2015;61(3):135 141. (10.1016/j.jphys.2015.05.014) 26093810

[17] Lombardi I Magri AG Fleury AM Da Silva AC Natour J . Progressive resistance training in patients with shoulder impingement syndrome: a randomized controlled trial. Arthritis Rheum. 2008;59(5):615 622. (10.1002/art.23576) 18438933

[18] Johnson MI . Resolving long-standing uncertainty about the clinical efficacy of transcutaneous electrical nerve stimulation (TENS) to relieve pain: a comprehensive review of factors influencing outcome. Medicina (Kaunas). 2021;57(4):378. (10.3390/medicina57040378) PMC8070828 33919821

[19] Desmeules F Boudreault J Roy JS Dionne CE Frémont P MacDermid JC . Efficacy of transcutaneous electrical nerve stimulation for rotator cuff tendinopathy: a systematic review. Physiotherapy. 2016;102(1):41 49. (10.1016/j.physio.2015.06.004) 26619821

[20] Rampazo ÉP Liebano RE . Analgesic effects of interferential current therapy: A narrative review. Medicina (Kaunas). 2022;58(1):141. (10.3390/medicina58010141) PMC8779694 35056448

[21] Chipchase LS Williams MT Robertson VJ . A national study of the availability and use of electrophysical agents by Australian physiotherapists. Physiother Theor Pract. 2009;25(4):279 296. (10.1080/09593980902782611) 19418365

[22] Van Der Heijden GJ Leffers P Wolters PJ , et al. No effect of bipolar interferential electrotherapy and pulsed ultrasound for soft tissue shoulder disorders: a randomised controlled trial. Ann Rheum Dis. 1999;58(9):530 540. (10.1136/ard.58.9.530) 10460185 PMC1752938

[23] Simpson PM Fouche PF Thomas RE Bendall JC . Transcutaneous electrical nerve stimulation for relieving acute pain in the prehospital setting: a systematic review and meta-analysis of randomized-controlled trials. Eur J Emerg Med. 2014;21(1):10 17. (10.1097/MEJ.0b013e328363c9c1) 23839103

[24] Johnson MI Jones G . Transcutaneous electrical nerve stimulation: current status of evidence. Pain Manag. 2017;7(1):1 4. (10.2217/pmt-2016-0030) 27641909

[25] Rani P Kalyani V Goyal T Yadav R Mishra R . Effect of transcutaneous electrical nerve stimulation therapy on pain and functional disabiliity level among patients with rotator cuff disease-a randomized controlled trial. Int J Physiother. 2020;7(1):7 13.

[26] Dingemanse R Randsdorp M Koes BW Huisstede BM . Evidence for the effectiveness of electrophysical modalities for treatment of medial and lateral epicondylitis: a systematic review. Br J Sports Med. 2014;48(12):957 965. (10.1136/bjsports-2012-091513) 23335238

[27] Kroeling P Gross A Graham N , et al. Electrotherapy for neck pain. Cochrane Database Syst Rev. 2013;8:CD004251. (10.1002/14651858.CD004251.pub5) PMC10696490 23979926

[28] Green S Buchbinder R Hetrick S . Physiotherapy interventions for shoulder pain. Cochrane Database Syst Rev. 2003;2003(2):CD004258. (10.1002/14651858.CD004258) 12804509 PMC8769566

